# Prevalence of traded bird species in key biodiversity areas

**DOI:** 10.1111/cobi.70277

**Published:** 2026-03-30

**Authors:** Oscar Morton, Tom Lansley, Stuart H. M. Butchart, Graeme M. Buchanan, David P. Edwards

**Affiliations:** ^1^ Department of Plant Sciences and Centre for Global Wood Security University of Cambridge Cambridge UK; ^2^ Conservation Research Institute University of Cambridge Cambridge UK; ^3^ School of Biosciences University of Sheffield Sheffield UK; ^4^ BirdLife International Cambridge UK; ^5^ Department of Zoology University of Cambridge Cambridge UK; ^6^ RSPB Edinburgh UK

**Keywords:** key biodiversity areas, protected areas, threatened species, trade, wildlife trade, Áreas clave para la biodiversidad, áreas protegidas, comercio de vida silvestre, especies amenazadas

## Abstract

The use and trade of biodiversity involve tens of thousands of species that are exploited at a range of scales, intensities, and degrees of sustainability. As a result, some are highly threatened. Key biodiversity areas (KBAs) are sites of significance for the persistence of biodiversity identified nationally based on standardized criteria. The degree to which biodiversity in KBAs is likely to be used and traded is not well understood. We undertook a fine‐scale assessment of the area of habitat (AOH) of traded species in KBAs. We used data on >16,000 KBAs and combined these with AOH maps for >10,000 bird species, a heavily traded and well‐documented indicator taxon. We subsequently mapped and quantified the distribution of traded species richness, extinction risk, and range‐size rarity in and adjacent to KBAs. Although less than half of all bird species are traded, we found that most bird species in KBAs were traded, and the richness of traded species was more than twice (119%) that of nontraded species. Traded species had comparable proportions of their AOH (22.5% on average) in KBAs as nontraded species (21.5%). Global hotspots of richness, extinction risk, and range rarity for traded species disproportionately occurred in KBAs, which had higher levels of extinction risk and range rarity for traded species than locations immediately outside KBAs. Of the 46 species that are likely threatened by unsustainable use and have the majority of their habitat falling within KBAs, we found that eight species had no habitat in designated protected areas. Reconciling sustainable use and the critical importance of KBAs for threatened species is essential. Our results suggest that for some species, there is an urgent need to protect key sites and ensure that any use and trade of species within them are sustainable.

## Abstract (es)

Prevalencia de especies de aves objeto de comercio en áreas clave para la biodiversidad

**Resumen:**

El uso y el comercio de la biodiversidad abarcan decenas de miles de especies que se explotan a diversas escalas, intensidades y niveles de sostenibilidad. Como resultado, algunas se encuentran gravemente amenazadas. Las áreas clave para la biodiversidad (ACB) son sitios de importancia para la persistencia de la biodiversidad, identificados a nivel nacional con base en criterios estandarizados. Aún no se conoce bien en qué medida es probable que se utilice y comercialice la biodiversidad en las ACB. Llevamos a cabo una evaluación a escala detallada del área de hábitat de las especies objeto de comercio en las ACB. Utilizamos datos de más de 16 000 ACB y los combinamos con mapas del área de hábitat (AOH) de más de 10 000 especies de aves, un taxón indicador muy comercializado y bien documentado. Posteriormente, cartografiamos y cuantificamos la distribución de la riqueza de especies objeto de comercio, el riesgo de extinción y la rareza del tamaño del área de distribución dentro de las ACB y en sus alrededores. Aunque menos de la mitad de todas las especies de aves son objeto de comercio, observamos que la mayoría de las especies de aves presentes en las ACB eran objeto de comercio, y que la riqueza de especies comercializadas era más del doble (119 %) que la de las especies no comercializadas. Las especies comercializadas tenían proporciones comparables de su área de distribución (AOH) (22,5 % en promedio) en las ACB que las especies no comercializadas (21,5 %). Los puntos críticos globales de riqueza, riesgo de extinción y rareza de distribución para las especies comercializadas se concentraban de manera desproporcionada en las ACB, que presentaban niveles más altos de riesgo de extinción y rareza de distribución para las especies comercializadas que las ubicaciones inmediatamente fuera de las ACB. De las 46 especies que probablemente se vean amenazadas por un uso insostenible y cuyo hábitat se encuentra en su mayor parte dentro de las ACB, observamos que ocho especies no tenían hábitat en áreas protegidas designadas. Es fundamental conciliar el uso sostenible con la importancia crítica de las ACB para las especies amenazadas. Nuestros resultados sugieren que, para algunas especies, existe una necesidad urgente de proteger los sitios clave y garantizar que cualquier uso y comercio de las especies que allí habitan sea sostenible.

## INTRODUCTION

Overexploitation is a key driver of the global biodiversity crisis (Jaureguiberry et al., 2022; Maxwell et al., 2016). Estimates posit that 52% of intact tropical forest is at least partially devoid of large mammals (Benítez‐López et al., 2019) and at least 24% of vertebrates are traded in some form (henceforth *traded species*), with this rising to 45% in birds (Donald et al., 2024; Scheffers et al., 2019). Ensuring that the take, and hence the trade, of these species is sustainable, particularly within area‐based conservation efforts, is therefore essential (Maxwell et al., 2020).

The sustainable use and trade of species have the potential to benefit conservation efforts substantially by incentivizing conservation interventions, aiding population recovery, and providing critical livelihoods and income for millions of people around the world (Hutton & Leader‐Williams, 2003; Nielsen et al., 2018; ’t Sas‐Rolfes et al., 2022). Yet, where offtakes are unsustainable or poorly managed, exploitation can drive steep population declines (Benítez‐López et al., 2017; Hughes et al., 2023; Morton et al., 2021). Of threatened (vulnerable, endangered, and critically endangered) and near‐threatened species (as classified on the International Union for Conservation of Nature [IUCN] Red List), ∼28% are likely experiencing elevated extinction risk due to intentional biological resource use, including hunting, fishing, and logging (Marsh et al., 2022).

Hunting and use are prevalent across global biodiversity hotspots (Harrison et al., 2016) and often interact synergistically with other key threats, including habitat loss, to trigger population declines (Symes et al., 2018). Even in protected areas (PAs), traded or hunted populations can still be subject to substantial population declines (Benítez‐López et al., 2019; Morton et al., 2021; Rija et al., 2020), although in some cases, these declines may be lower than would be expected in the absence of protection (Benítez‐López et al., 2017).

Key biodiversity areas (KBAs) are sites of significance for the global persistence of biodiversity and are identified nationally based on quantitative criteria relating to threatened or geographically restricted species or ecosystems, biological processes, ecological integrity, and irreplaceability (IUCN, 2016). To date, >16,500 sites have been identified as KBAs. They span all countries and cover >22 million km^2^ of terrestrial, freshwater, and marine environments (BirdLife International, 2024). These critical areas for biodiversity face stressors, including low forest integrity (Crowe et al., 2023), infrastructure development (Simkins et al., 2023), climate change (Butchart et al., 2021), and fragmentation and human disturbance (Yang et al., 2024). Past and recent analyses also highlight the growing human population in biodiversity hotspots globally (Cincotta et al., 2000; Cunningham & Beazley, 2018). Concurrently, roads, power stations, mines, and other associated infrastructure are projected to increase in KBAs globally (Simkins et al., 2023). This increasing human footprint and accessibility in and around KBAs further necessitate consideration of the occurrence and prevalence of the use and trade of species in KBAs to ensure both the effective conservation of used species and the maintenance of critical livelihoods. Global‐scale patterns of risk associated with hunting or trade are inherently coarse and poorly quantified because unlike landcover or climatic changes, use and trade impacts cannot be detected from remote sensing or be delineated to specific areas of species ranges at scale. Inference based on the limited number of local‐level studies suggests that levels of hunting, subsistence use, and trade in KBAs can be a significant concern (BirdLife International, 2014; Pattimahu & Kastanya, 2021; Smiley Evans et al., 2020).

Globally, birds are broadly well represented in the KBA network (Lansley et al., 2025) and are proportionally the most traded vertebrate group (Donald et al., 2024; Scheffers et al., 2019). Trade is a key driver of threat for many bird species and triggered the aptly named Asian songbird crisis (Lees & Yuda, 2022). We assessed the importance of KBAs for traded species with three objectives in mind: determine whether traded species are proportionally represented globally in KBAs; determine whether KBAs have higher traded species diversity (richness, extinction risk, and range‐size rarity) than areas outside KBAs; and assess how comprehensively use and trade‐threatened species primarily restricted to KBAs are covered by existing protection to better identify gaps. We hypothesized two alternative relationships between traded avian richness and KBAs, which we aimed to test at scale. First, traded species richness is lower in KBAs because habitat degradation and exploitation outside KBAs deplete populations of traded species, rendering them less likely to meet KBA criteria. Second, as a product of the KBA assessment criteria (IUCN, 2016), KBAs host hotspots of threatened or range‐restricted species (Eken et al., 2004), which prior studies highlight are proportionally more likely to be traded (Scheffers et al., 2019). To discern this spatial pattern of traded species occurrence in KBAs, we also assessed two complementary measures of diversity capturing different facets of traded rarity: species’ extinction risk (threat‐weighted species richness), which accounts for the presence of increasingly threatened species, and range‐rarity‐weighted richness, which gives greater weight to species with smaller ranges. These additional metrics relate directly to the qualification criteria used to determine KBA status (IUCN, 2016) and allowed us to better ascertain more complex drivers of traded diversity in KBAs, such as whether comparatively range‐restricted or threatened species drive patterns in traded species representation in KBAs rather than richness alone.

## METHODS

### Quantifying traded species’ area of habitat

We identified likely traded species following Scheffers et al. (2019), who drew on data from the Convention on International Trade in Endangered Species of Wild Fauna and Flora, and the IUCN Red List. We extracted the list of all traded bird species and matched this to the current BirdLife International taxonomy (HBW & BirdLife International, 2024), which provides the basis for IUCN Red List assessments for birds.

We used recently produced area of habitat (AOH) maps (Brooks et al., 2019) for all bird species (Lumbierres et al., 2022). Maps were projected to Behrmann equal area cylindrical projection with a spatial resolution of 100 m cells. The AOH maps are used to estimate the distribution of suitable habitat within a species’ range, improving the accuracy of conservation inference and reflecting where species are likely to occur within their documented range limits (Lumbierres et al., 2022). We matched species’ AOH maps with the list of species classed as traded or not traded per Scheffers et al. (2019). This resulted in a total of 3511 resident species and 845 migratory species with separate breeding and nonbreeding AOH maps. We merged the latter to create a single AOH map per species (totaling 4356 traded species with corresponding AOH maps).

### Quantifying species’ occurrence in KBAs

The boundaries of 16,012 KBAs were accessed from BirdLife International (2022). As per Lansley et al. (2025), KBA polygons were reprojected to a Behrmann equal area cylindrical projection identical to the species’ AOH rasters. Overlapping KBA polygons were dissolved to remove overlaps. Polygons with invalid, self‐intersecting geometries were dissolved. Nine KBA polygons were not solved, with their geometries remaining invalid, and were removed from further analyses. As per Lansley et al. (2025), we created a data matrix with each combination of KBA and AOH. The matrix included the species’ AOH in each KBA. Each AOH map was cropped and masked by the extent of the KBA file with R 4.4.2 (R Core Team, 2024) and the raster 3.6‐3.2 (Hijmans, 2023) and sf 1.0‐21 packages (Pebesma, 2018).

We fitted two models to test whether the richness of traded species inside KBAs was greater than that of nontraded species and whether traded and nontraded species differed in the proportion of their AOH that was inside KBAs. The first model was a count model assuming a negative binomial error distribution that estimated species richness as a function of KBA area (log10 transformed and standardized) and a binary indicator variable indicating traded or nontraded species richness. We included the hierarchical effect of region (Asia, Africa, Europe, Oceania, and North and South America) and allowed this to vary between traded and nontraded groups.

The second model was an ordered beta binomial model, reflecting the proportion of a species’ AOH in KBAs ranges from 0 to 1 (inclusive) (Kubinec, 2023). This included species’ AOH extent (log_10_ transformed and standardized) and a binary indicator of whether a species was traded or not as covariates. We included the hierarchical effect of species’ taxonomic order and allowed this effect to vary between traded and nontraded species. All models were fitted in R with brms 2.18.0 (Bürkner, 2017), and models were run for 500 warm‐up and 500 sampling iterations. All priors were specified as zero‐centered and diffuse, reflecting little a priori evidence of a directional effect and allowing estimates to be primarily informed by the data. All models were checked to ensure stable convergence with the *R*‐hat (Gelman–Rubin) statistic (<1.05 denoting sufficient between‐chain convergence). Subsequent posterior predictive checks were used to ensure the models recovered key features of the fitting data. All postfitting processing used tidybayes 3.0.7 (Kay, 2020) and bayestestR 0.13.0 (Makowski et al., 2019). We used the probability of direction (pd) to ascertain the certainty of an association's direction. We considered a pd of >97.5% as significant evidence of directionality or difference. Across analyses, we summarized posterior distributions to the median and the 70% and 90% highest density intervals (HDIs).

### Potential occurrence of traded species inside and outside KBAs

We calculated three metrics of traded species’ global distribution: species alpha richness, extinction risk, and range‐size rarity. With these complementary metrics, we aimed to discern differences between broad patterns of species distributions and the specific facets of rarity that map directly onto KBA assessment criteria (e.g., extinction risk and range size). Traded alpha richness was calculated by stacking the AOH maps of all species to obtain the total number of traded species per 100‐m cell.

Extinction risk was calculated as the mean threat score (T) of all species occurring in a given cell (n). This was defined using species’ IUCN Red List categories following a geometric progression: 2, least concern; 4, near threatened, 8, vulnerable; 16,   endangered; 32, critically endangered (Sze et al., 2024; Wang et al., 2020). We calculated extinction risk as

(1) $$\frac{\sum_{i=1}^{n}T_{i}}{n}.$$

We recalculated extinction risk with an alternate scoring system (0, least concern; 1, near threatened; 2, vulnerable; 3, endangered; 4, critically endangered) based on the equal‐steps relative extinction risk used in the IUCN Red List criteria. The results of this are in the Supporting Information, and the results aligned closely with those based on the geometric weighting.

Range‐size rarity reflects concentrations of species with a small area of suitable habitat (Sze et al., 2024; Veach et al., 2017). This is calculated as the sum of the inverse area of habitat ($\mathrm{AO}H_{i}$) for all species occurring in a cell divided by the total number of species occurring in the cell (n). A maximum value of 1 would denote that the cell contains the total AOH for all species occurring in that cell.

(2) $$\mathrm{Range}-\mathrm{size}\;\mathrm{rarity}=\frac{\sum_{i=1}^{n}\frac{1}{\mathrm{AO}H_{i}}}{n}$$

We then identified hotspots as areas with values in the highest 5th and 25th percentiles. We compared the proportion of these hotspots that fell into KBAs with the proportion that would be expected at random based on the proportion of the global layer (e.g., excluding the poles) that KBAs cover.

To assess whether areas inside KBAs contained higher values of our metrics of traded species (traded species’ richness, extinction risk, and range‐size rarity) than outside KBAs, we buffered each KBA by 10 km. The metrics inside KBAs were then compared with the values in the 10‐km buffer, following Sze et al. (2024). This approach was chosen over spatial matching as matching pixels is highly unlikely to effectively identify all possible covariates that drive local‐scale patterns of species’ distributions and wider‐scale patterns of species richness (Sze et al., 2024). However, our contrasting approach, comparing metrics both inside and outside KBAs, does allow inference of what likely difference there is. This is not designed to determine causality (e.g., if KBAs drive increases in traded species richness) but to contextualize the importance of KBAs relative to the wider landscape.

To quantify the difference between KBAs and their 10‐km buffer, we carried out country‐level permutation tests. First, null distributions were created per country, where pixels equal to the number of pixels inside the KBA (pseudo‐KBA) and in the 10‐km buffer area (pseudobuffer area) were sampled. The difference was then calculated between the average pseudo‐KBA area and pseudobuffer area. This was repeated 1000 times per country to create country‐level null distributions. Then, the observed difference between KBAs and the buffer was calculated per country. The null distribution was then used to calculate *p* values with a two‐tailed test. To account for the risk of inflated type I error rate, *p* values were adjusted using the conservative Holm method, and subsequent significance was classed as (α < 0.05). We repeated this in the supplement with a less‐conservative but more confirmatory Benjamini–Hochberg false discovery rate correction, which yielded almost identical inference (Appendix S3).

A supplementary comparative analysis of nontraded species hotspots and potential occurrence in‐ and outside KBAs (with a 10‐km buffer) was also carried out to assess the extent to which traded species had globally different patterns of overlap in KBAs than nontraded species. This was implemented for all three calculated metrics: species richness, extinction risk, and range‐size rarity. For computational reasons, these supplementary analyses were carried out at a 1‐km spatial resolution (Appendices S4 & S5).

### Extent of protection for range‐restricted species threatened by biological resource use

Following Marsh et al. (2022), we used IUCN Red List data on threats to identify species for which biological resource use is documented as a threat. In some cases, species may be threatened by use but not trade (because they are threatened by subsistence use, with no or little trade). However, given that trade first requires the extraction (hunting or trapping) of individuals, it is likely a reasonable proxy. Specifically, we determined which near‐threatened and threatened (vulnerable, endangered, and critically endangered) species are recorded as affected by threat “5.1 biological resource use: hunting and collecting terrestrial animals” (we excluded threats 5.2–5.4, which primarily concern use of plants, logging, and fisheries). Within 5.1, we further excluded unintentional use (5.1.2) and persecution or control (5.1.3). We retained only 5.1.1 intentional use or 5.1.4 unknown. We included the unknown category as a precaution to reflect that understanding of motivations is often incomplete. Following the IUCN–BirdLife methodology and Marsh et al. (2022), we used an additive scoring system to account for the threats’ scope, timing, and severity so that the score was greatest for species undergoing rapid declines driven by the threat over its entire range. Only those species that had an overall medium or high threat impact score (a combined scope, timing, and severity score >6) were considered threatened by biological resource use (Appendix S6). However, within a species’ distribution, it is possible for local populations to be severely declining due to local pressures, despite stable or positive overall trends; likewise, even where declines are widespread and rapid, it is possible that these effects may not be reflected in IUCN Red List assessments for some years (Kamp et al., 2015).

We then extracted all species that are likely threatened by biological resource use and that had the majority (at least 50%) of their AOH in KBAs (total *n* = 46). We assessed the representation of these species in PAs based on the assumption that species are sufficiently protected if 100% of AOHs <1000 km^2^ fall within PAs and 10% of AOHs >250,000 km^2^ fall within PAs, with coverage log‐linearly interpolated for intermediate values following Hanson et al. (2020). For these species, we calculated the extent of their AOH that fell within unprotected KBAs, protected KBAs, or PAs outside KBAs based on information in the World Database of Protected Areas (UNEP‐WCMC & IUCN, 2024). We kept only those PAs whose status was classed as “designated,” “inscribed,” or “established” (i.e., excluding “proposed” and “degazetted”). We removed marine areas and areas for which the status was “not applicable.”

## RESULTS

### KBA coverage of traded species

**FIGURE 1:**
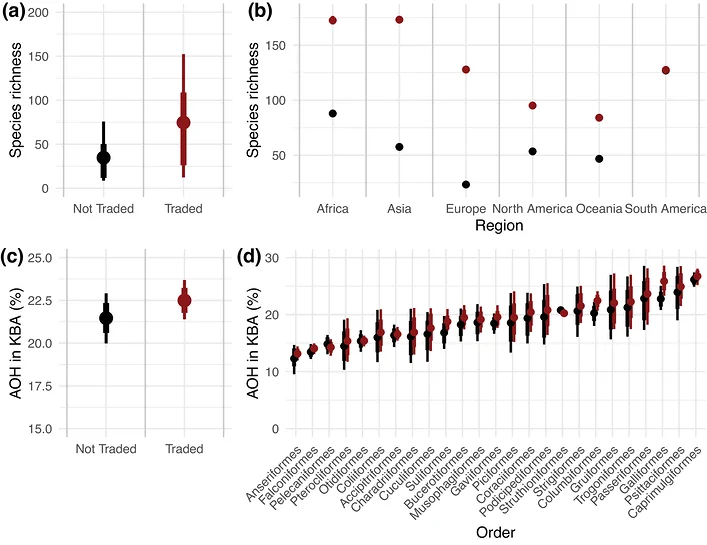
Average number of traded and untraded bird species with area of habitat (AOH) inside key biodiversity areas (KBAs) in (a) total and (b) by region and average percentage of AOH inside KBAs (c) for traded and untraded species and (d) for the average species of each taxonomic order (points, posterior medians; thick bars, 70% highest density interval; thin bars, 90% highest density interval).

Of the 14,966 KBAs (93.5% of analyzed KBAs) that overlapped with the terrestrial AOH for at least one bird species, 92.5% of these KBAs (13,849) contained more traded than nontraded species. This is despite the finding that only 41.0% of species included in our analyses were traded. Most contained traded and nontraded species, but 90 KBAs contained only traded species, whereas 12 contained only nontraded species. On average, KBAs were estimated to have 119.4% (90% HDI 26.3–235.7%, pd = 98.65%) more traded than nontraded species, although there was considerable variation around this estimate (Figure 1a). This pattern held in KBAs across Africa, Asia, Europe, North America, and Oceania. The number of traded species on average was 78.1% (North America) to 447.4% (Europe) greater than the number of nontraded species (Figure 1c). Only South America deviated from this trend, where on average, KBAs had no clear difference in traded or nontraded species richness. We did not detect a difference (pd = 93.60%) in the average percentage of species’ AOH that fell within KBAs for traded or untraded species (Figure 1b). This was the case across taxonomic orders (Figure 1d). In supplementary analyses, there was a clear difference in the areal extent of AOH inside KBAs between traded and nontraded species; traded species had 248.0% (188.8–305.7%) more area inside KBAs than those not traded (Appendix S1).

**FIGURE 2:**
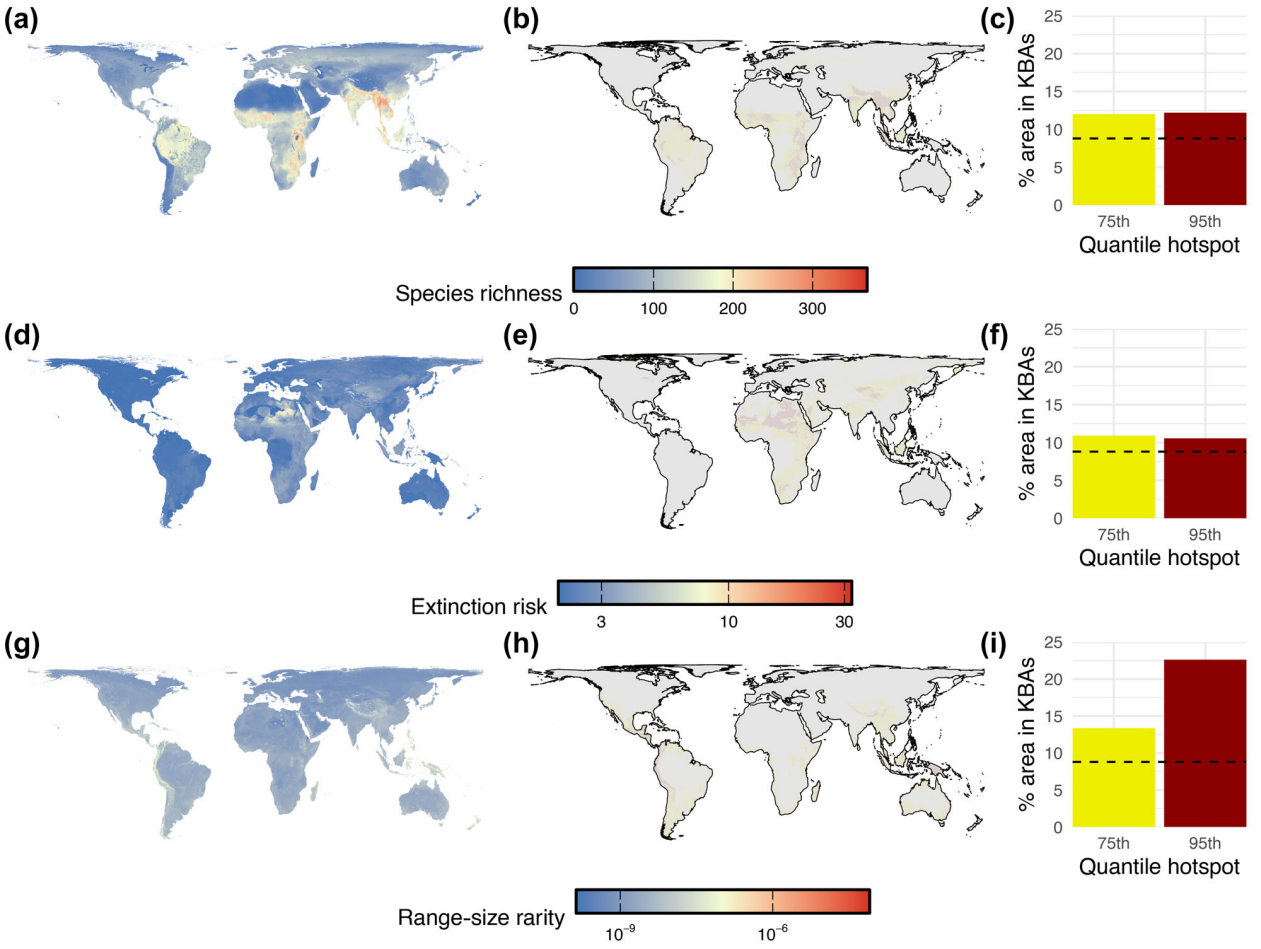
Traded species’ (a) richness and (b) richness hotspots (brown, 5th percentile; yellow, 25th percentile), (c) percentage of the extent of hotspots in KBAs (dashed lines, 8.8%, which is the proportion of the terrestrial area globally covered by KBAs [i.e., coverage that could be expected if hotspots were randomly distributed relative to KBAs]; dark red, 5th percentile; yellow, 25th percentile), and percentage of the extent of hotspots in KBAs for (d–f) extinction risk and (g–i) range‐size rarity.

Use of high‐resolution species’ AOH maps rather than range maps revealed hotspots of traded species’ diversity obscured in previous work. The Andean region across Colombia, Ecuador, Peru, and Bolivia (highlighted by Scheffers et al. [2019]) did not appear as a key hotspot (defined as above the 95th quantile of values) with our approach (Figure 2a,b). This was likely due to the exclusion of many species from cells once range maps were cropped by species’ elevational limits and habitat preferences to generate AOH maps. Other richness hotspots across sub‐Saharan Africa and Southeast Asia highlighted by Scheffers et al. (2019) were also identified, although they appeared more fragmented, reflecting the greater resolution and ecological nuance of AOH maps.

Hotspots of extinction risk for traded species were primarily in areas of low traded species’ richness (Saharan Africa, eastern China, etc.), where the presence of even a single highly threatened traded species could highlight the region (Figure 2d,e). A small number of extinction‐risk hotspots occurred in areas rich in traded species across Ethiopia, Kenya, Tanzania, Zambia, Indonesia, Malaysia, and the Philippines. These patterns remain consistent even when weighting extinction risk based on equal‐step scores for each IUCN Red List category (Appendix S2). Conversely, hotspots of traded species’ range‐size rarity were far more widely distributed and were concentrated along the Andes and Central America, Madagascar, Papua New Guinea, Indonesia, the Philippines, and small island nations, including the Solomon Islands and Fiji (Figure 2g,h).

Across all metrics (species richness, extinction risk, and range‐size rarity), hotspots of traded species occurred disproportionately inside KBAs (Figure 2c,f,i). This was most evident for traded species’ range‐size rarity, with 22.6% of the top 95th quantile hotspots occurring in KBAs. A total of 12.0% and 12.2% of species’ richness hotspots and 10.9% and 10.6% of extinction risk hotspots occurred inside KBAs, respectively, for the 75th and 95th quantiles. These patterns were similar for nontraded species’ richness and range‐size rarity but not for nontraded species’ extinction risk, which fell just below what would be expected at random (Appendix S4).

### Importance of KBAs for threatened species and their habitat

**FIGURE 3:**
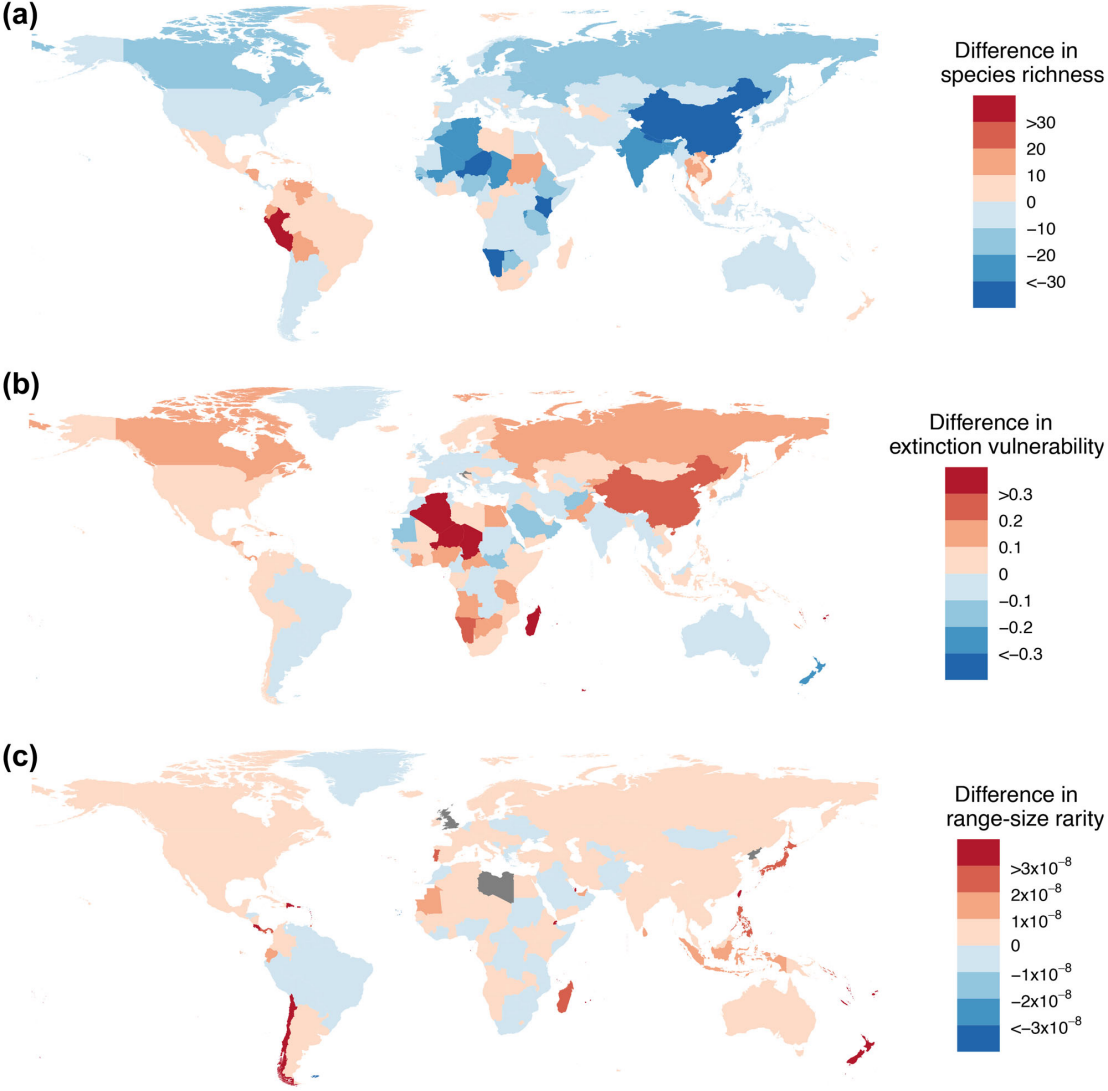
Difference between traded bird species’ (a) richness, (b) extinction risk, and (c) range‐size rarity inside key biodiversity areas (KBAs) and in 10‐km buffers surrounding them (red tones, higher values inside KBAs; blue tones, higher values outside KBAs; gray, no significant difference at α = 0.05 [Holm adjusted] between values inside and outside KBAs).

On average, KBAs had lower traded species’ richness than their 10‐km buffer zone in 66.8% of assessed countries and territories (143 of 214 at *p* < 0.05). The greatest differences occurred in mainland China, Niger, and Namibia, where on average KBAs had 39, 58, and 69 fewer traded species, respectively, than the surrounding area (Figure 3a). However, contrasting trends occur in 68 territories (at *p* < 0.05), particularly across South America and in small island states. The greatest observed difference occurred in Peru and Nicaragua, where on average 42 and 19 more traded species were found in KBAs than the surrounding areas.

The global pattern was broadly reversed for extinction risk and range‐size rarity as KBAs generally had significantly higher values of extinction risk (62.1%, 133 of 214, at *p* < 0.05) and range‐size rarity (68.7%, 147 of 214, at *p* < 0.05) for traded species than the surrounding area (Figure 3b). Territories where KBAs hosted the highest levels of traded species’ extinction risk relative to the surrounding area were Seychelles, Singapore, São Tomé and Príncipe, and Mauritius. Similarly, KBAs in Tonga, Montserrat, Mauritius, Palau, and São Tomé and Príncipe, again a group of countries dominated by islands, had substantially greater range‐size rarity values for traded species than the surrounding buffer area. A number of island territories (including Seychelles, Antigua and Barbuda, and St Martin) and larger nations, including Angola, Somalia, Greenland, and Peru, deviated from this pattern and had significantly lower range‐size rarity inside KBAs than the surrounding buffer (Figure 2c).

Patterns of greater species’ richness outside KBAs were weaker but remained for nontraded species’ richness (55.4% of assessed countries and territories at *p* < 0.05, compared with 66.8% for traded species) (Appendix S5). However, patterns of extinction risk and range rarity differed more substantially between traded and nontraded species (Appendices S4 & S5). Extinction risk of nontraded species was greater in KBAs in only 30.0% of countries and territories (compared with 62.1% for traded species). This was largely due to the number of countries and territories that had comparable values of extinction risk of nontraded species in‐ and outside KBAs (31.9% for nontraded species relative to 2.3% for traded). Similarly, nontraded range rarity was significantly greater in only 51.6% of countries and territories (compared with 68.7% for traded species).

### Conserving used species and KBAs

**FIGURE 4:**
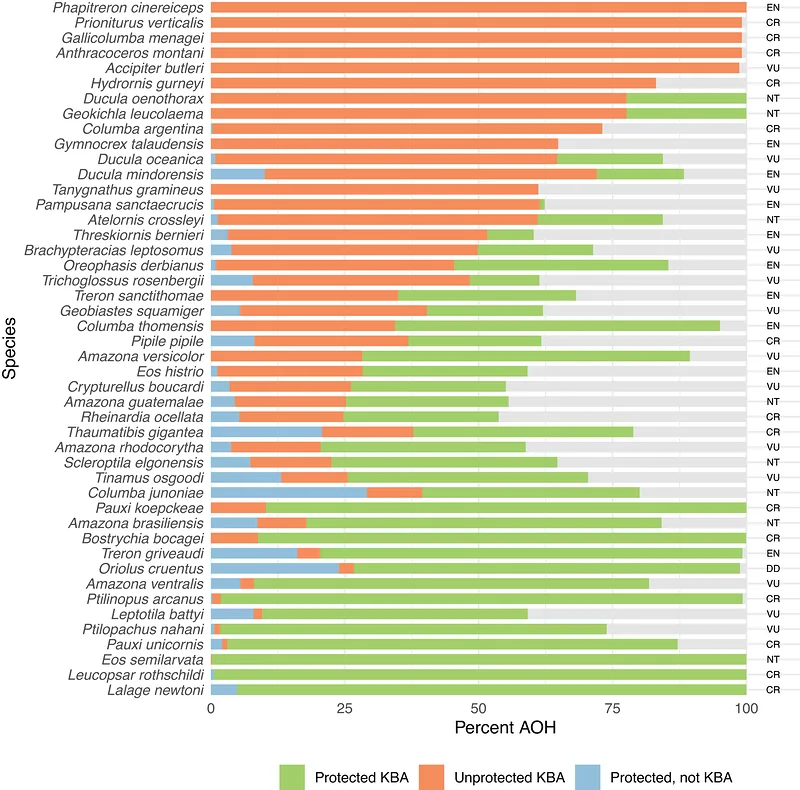
Protected area coverage of likely unsustainably used species with >50% of their area of habitat (AOH) in key biodiversity areas (KBAs) (gray, areas not in KBAs or protected areas; CR, critically endangered; EN, endangered; VU, vulnerable; NT, near threatened; LC, least concern; DD, data deficient; species ordered by percentage of AOH that is unprotected and in KBAs; common names in Appendix S7).

Forty‐six traded species had the majority (>50%) of their AOH inside KBAs and were likely threatened by biological resource use and as such are of conservation concern (Figure 4). Most of these species (82.6%, 38 or 46) had some proportion of their total AOH in PAs; however, this ranged from 0.3% to 100% (mean = 52.3%). Eight species likely threatened by unsustainable use and predominantly occurring in KBAs did not overlap with any PAs. These species all had relatively small ranges (AOH 20.3 – 1031.6 km^2^, mean = 486.9 km^2^).

Only 17.4% (8 out of 46) of likely unsustainably used species were sufficiently covered by PAs. Of the 38 species lacking sufficient protection, the majority (22 species) had highly restricted AOHs (<1000 km^2^). When we considered a scenario in which all KBAs became protected in the future, the number of species with insufficient coverage of their AOH by PAs declined from 38 to 26.

This result highlights a number of likely unsustainably used species in KBAs that occurred exclusively in protected KBAs (Figure 4). These included two species found only in Indonesia: Bali myna (*Leucopsar rothschildi*) (which is highly, negatively affected by collection for the national and international cagebird trade and largely restricted to a release site in Bali Barat National Park) and the highly range‐restricted blue‐eared lory (*Eos semilarvata*) (thought to be locally threatened by collection and export [Nandika et al., 2021]).

## DISCUSSION

The most important sites for biodiversity globally, KBAs, are disproportionately important for the conservation of traded species. We found that global hotspots of traded bird richness, extinction risk, and range‐size rarity disproportionately occurred in KBAs. On average, KBAs hosted more than twice the number of traded species than untraded species. In most countries, traded bird richness was lower in KBAs than the surrounding habitat, but traded bird species extinction risk and range‐size rarity was greater inside KBAs than the surrounding habitat for most territories. This difference was particularly large for traded bird extinction risk relative to nontraded species’ extinction risk. This emphasizes the importance of conserving KBAs for threatened traded species. It is important to implement protection policies and actions to ensure that any use of species in KBAs is sustainable, particularly for the 46 threatened or near‐threatened species specifically threatened by biological resource use that are largely restricted to KBAs.

### Importance of KBAs for traded species’ habitat

The observed predominance of used species compared with nonused species in KBAs was likely due to the positive correlation between species’ range size and their occurrence in trade (Donald et al., 2024). For traded species, the generally lower levels of species richness but greater extinction risk and range‐size rarity inside KBAs than outside reflected the important role KBAs can play in conserving threatened and small‐ranged species (Figure 4). Thus, where high levels of use or trade of species within KBAs occurred, effective management is needed to prevent illegal use and ensure that any legal offtake is sustainable, and management approaches do not adversely affect local livelihoods. Balancing the importance of well‐managed use as a conservation tool with the risk of poorly managed use driving negative impacts on species and populations is a preeminent challenge (Benítez‐López et al., 2017; Morton et al., 2021).

A global review of recreational hunting—a single type of use—highlighted the significant research bias toward large mammals in North America, Europe, and Africa. Only one‐quarter of studies concerned birds, and the 12 most‐studied species included only one bird species (mallard [*Anas platyrhynchos*]) (Di Minin et al., 2021). This study highlighted the overall lack of quantitative evidence on socioeconomic and conservation outcomes in most studies and systems (Di Minin et al., 2021). This issue is compounded for other uses (e.g., capture of wild birds for pets and the songbird trade), where there is an even poorer understanding of overall impacts (and for many species, what levels of use would be sustainable), despite the scale and ubiquity of harvest for the cagebird trade in certain regions (e.g., Indonesia) (Marshall et al., 2020). Of the 140 avian case studies involving hunting and trapping of live individuals available on the IUCN Species Use Database, only two are considered sustainable and both refer to past rather ongoing exploitation. In 41 case studies, levels of use are considered unsustainable, with sustainability being unknown or unreported for 97 (IUCN, 2024).

### Effective protection for species and sites

Using KBAs—with some additional areas—to prioritize the expansion of the global PA network would be effective in capturing global biodiversity (Kullberg et al., 2019). Prioritization exercises focus on optimizing conservation metrics to identify important areas for protection (Kullberg et al., 2015; Plumptre et al., 2024). Given the relatively high levels of used species’ occurrence in KBAs, integrating conservation goals with species’ management plans that permit a level of sustainable use may be necessary for species with a low risk of extinction. Multiuse and sustainable‐use PAs have the potential for cobenefits to species and communities, provided they are well managed (e.g., sustainable‐use PAs report significantly higher socioeconomic outcomes than strict PAs) (Campos‐Silva et al., 2021; Oldekop et al., 2016). There is growing recognition of the importance of local communities and Indigenous Peoples for conserving species and their habitat (Sze et al., 2022, 2024). Unprotected KBAs inside Indigenous Peoples’ lands experience lower levels of tree cover loss than unprotected KBAs outside Indigenous Peoples lands (although levels of tree cover loss in PAs average far lower [Simkins et al., 2024]). Given the critical importance of used species for rural communities globally (Belant et al., 2024; Nielsen et al., 2017), involving the people living in and around KBAs in their conservation—particularly for KBAs with high numbers of used species—is key for achieving long‐term biodiversity outcomes.

In specific cases, well‐managed, strict no‐take PAs may be the only way to preserve heavily used and threatened species with dwindling populations and a high risk of extinction (Cazalis et al., 2020). For example, the critically endangered dwarf ibis (*Bostrychia bocagei*), restricted to São Tomé and Príncipe, is heavily threatened by hunting at levels estimated to be unsustainable, particularly given that the global population may number as few as 130 mature individuals (BirdLife International, 2020b). Its remaining AOH falls entirely within KBAs, with a substantial proportion of this within the São Tomé Obô Natural Park. Local initiatives are being developed for ecotourism and to train local people as bird guides (BirdLife International, 2020b; Lima et al., 2017). However, even for relatively large charismatic species, protection is not always effective, with the critically endangered horned curassow (*Pauxi unicornis*) occurring almost entirely in PAs in Bolivia (Amboró and Carrasco National Parks and the Isiboro‐Secure Indigenous Territory and National Park). However, these PAs are subject to encroachment by human settlement and unsustainable levels of hunting (BirdLife International, 2016a; Killeen et al., 2007). For unprotected, highly range‐restricted, threatened species that are used, such as the Sulu racquet‐tail (*Prioniturus verticalis*) and the Sulu hornbill (*Anthracoceros montani*) (both critically endangered species found only in the Sulu archipelago of the Philippines), strict protection of their little remaining habitat is essential to prevent extinction. However, insurgency on Tawi Tawi island to which these species are restricted has posed a significant barrier to this (BirdLife International, 2016b, 2020a).

The expansion of the existing PA estate and implementation of effective management, including managing or prohibiting resource extraction such as hunting and trapping, must be coupled with engagement of local communities and provision of alternative livelihoods, where appropriate, to ensure positive outcomes for people and nature (Sandbrook et al., 2023). Likewise, in contexts in which conservation funding is limited, it may be more beneficial to threatened and exploited species to focus funding toward management of existing PAs than expanding the extent of PAs (Kuempel et al., 2018). Future research should focus on strengthening our understanding of the factors governing when use is sustainable. The majority of evaluations of use and trade in sustainable‐use PAs focus on species hunted for consumption, and focus predominantly on mammals, often finding mixed and context‐dependent results (depending on, e.g., community size and the nature of the remaining habitat matrix; Sampaio et al., 2023). Similarly, the relative dominance of studies from mainland sub‐Saharan Africa and Central and Southern America (Benítez‐López et al., 2017; Morton et al., 2021; Nielsen et al., 2017, 2018) risks omitting key hotspots of traded species extinction risk and range‐size rarity in small island states (e.g., Seychelles, São Tomé and Príncipe, Mauritius, Montserrat, Palau, Antigua and Barbuda, and St Martin) (Figure 3). Likewise, understanding the realized benefit of use is critical. In one study, in the Moramanga district of Madagascar, a biodiversity hotspot, collection of reptiles and amphibians for the international pet trade formed part of diverse livelihood strategies for many households but was perceived as unreliable and thus unlikely to incentivize protection and sustainable use (Robinson et al., 2018).

We used AOH to assess patterns of species’ distributions. Although AOH data have fewer commission errors than species’ range maps, they can fail to represent occupancy if species are absent due to historic biogeographical or recent anthropogenic factors (e.g., through negative impacts of alien invasive species or hunting). Additionally, AOH data rely on land‐cover maps derived from satellite remote sensing that themselves have errors and can be based on categories (e.g., temperate forest) that do not distinguish important differences that characterize species’ habitat (e.g., specific tree or plant habitat dependencies, such as the red‐cockaded woodpecker's [*Leuconotopicus borealis*] strong affinity for certain pine species). Likewise, global data on traded species also have limited spatial resolution. Species may not be used, collected, hunted, or traded across their entire range, and quantifying the intraspecific variation in use and trade within species’ ranges or AOH remains a significant research frontier. Our hotspot maps should be interpreted as hotspots of traded species’ AOH (or hotspots of traded species’ likely presence), not necessarily as hotspots of trade itself and not as hotspots of trade threat. Likewise, we used data from the IUCN Red List on threats from use, but the accuracy of these data relies on species’ assessments being up‐to‐date and comprehensive. Focusing on birds, for which all known species are typically assessed every 4 years, minimizes the risk of inaccurate or outdated information (Rondinini et al., 2014). However, as other taxa become increasingly well assessed in terms of distribution and exploitation, future work should develop risk assessments for overexploitation in KBAs for a greater diversity of species, as localized hotspots of risk likely differ between taxa (Jenkins et al., 2013).

In summary, KBAs are disproportionately important for used bird species and generally host substantially greater diversity of threatened and range‐restricted species that are used. Thus, their conservation is critical for ensuring a sustainable future for species used by people. Actions to conserve these sites and to ensure that use of species within them is sustainable and delivers lasting benefits to local people would contribute to multiple targets in the Kunming–Montreal Global Biodiversity Framework (including Targets 1, 3, 4, 5, 9, 10, and 22), particularly the need to concurrently conserve 30% of land, waters, and sea (Target 3) and manage wild species sustainably to benefit people (Target 9). Our findings emphasize the importance of considering the occurrence of used species when planning PA strategies and also the need to strictly protect KBAs that contain all or the majority of the habitat of a number of threatened and range‐restricted species that are currently heavily affected by unsustainable use.

## AUTHOR CONTRIBUTIONS

*Conceptualization*: Oscar Morton, David P. Edwards, Stuart H. M. Butchart, and Graeme M. Buchanan. *Data curation*: Oscar Morton and Tom Lansley. *Formal analyses*: Oscar Morton. *Writing—original draft*: Oscar Morton. *Writing—review and editing*: Oscar Morton, Tom Lansley, Stuart H. M. Butchart, Graeme M. Buchanan, and David P. Edwards.

## Supporting information



Supporting Information

## Data Availability

All data are from publicly available sources referenced in the methods text; code used for analysis is available at https://github.com/OMorton/KBAs_UseTrade.
