# Supplementary material for: Prevalence of traded bird species in key biodiversity areas
Source: Conserv Biol. 2026 Mar 30;40(5):e70277. doi: 10.1111/cobi.70277 (PMC13613543; doi:10.1111/cobi.70277)
Supplement: Supplementary file 1 [file COBI-40-e70277-s001.docx]

**Supplementary Materials**

 **Appendix S1. Hectares of AOH within KBAs for traded and untraded species.** Extent of AOH in KBAs for traded (red) and untraded species (black) for all species (A) and for taxonomic Orders (B). Points represent posterior medians and error bars show 90% HDIs.

 **Appendix S2. Maps and hotspots of extinction risk using an alternative approach of weighting Red List categories of extinction risk from 1 for Least Concern species to 4 for Critically Endangered species.** See Figure 2D, E and F for details.

**Appendix S3. Difference between traded species’ richness (A), extinction risk (B) and range-size rarity (C) inside Key Biodiversity Areas (KBAs) and inside 10-km buffers surrounding them.** Red tones denote higher values inside KBAs, blue tones denote higher values outside of KBAs, grey territories had no significant difference (at *α* = 0.05, Benjamini–Hochberg false discovery rate correction) between values inside and outside of KBAs.

**Appendix S4. Hotspots in the distribution of non-traded species and their occurrence in KBAs.** A. Not traded species richness hotspots (5^th^ and 25^th^ percentile shown in brown and yellow respectively). B. Percentage of the extent of hotspots occurring in KBAs (following the same colour scheme); the dashed reference line denotes 8.8%, which is the proportion of the terrestrial area globally covered by KBAs (i.e. the coverage that could be expected if hotspots were randomly distributed relative to KBAs). C and D show the same for extinction risk, and E. and F. for range-size rarity, respectively.

**Appendix S5. Difference in non-traded species’ richness (A.), extinction risk (B.) and range-size rarity (C.) inside and outside of KBAs.** Red tones denote higher values inside KBAs, blue tones denote higher values outside of KBAs, grey territories had no significant difference (at *α* = .05, holm-adjusted) between values inside and outside of KBAs.

**Appendix S6.** Threat scoring system as per the IUCN Red List (<https://www.iucnredlist.org/resources/threat-classification-scheme>) and adapted from Marsh et al. 2022.

| **Timing:** | | | | | *Ongoing threat (+3)* | | | *Future or past, likely to return threat (long term) (+1)* | | | | |
| --- | --- | --- | --- | --- | --- | --- | --- | --- | --- | --- | --- | --- |
| **Severity**: | *Very rapid*  (+3) | | *Rapid or unknown*  (+2) | | | *Slow or fluctuating*  (+1) | *Negligible or no impact* (0) | *Very rapid*  (+3) | *Rapid or Unknown*  (+2) | | *Slow or Fluctuating*  (+1) | *Negligible or no impact* (0) |
| **Scope** | |  | |  | |  |  |  | |  |  |  |
| *Whole (+3)* | | 9 | | 8 | | 7 | 6 | 7 | | 6 | 5 | 4 |
| *Majority or unknown (+2)* | | 8 | | 7 | | 6 | 5 | 6 | | 5 | 4 | 3 |
| *Minority (+1)* | | 7 | | 6 | | 5 | 4 | 5 | | 4 | 3 | 2 |

Additive impact scores classification as follows - 8-9: High impact threat, 6-7: Medium impact threat, 3-5: Low impact threat and 0-2: Negligible or no impact threat.

**Appendix S7.** Species binomial and common names in Figure 4.

| **Binomial** | **Common name** |
| --- | --- |
| *Lalage newtoni* | Reunion Cuckooshrike |
| *Leucopsar rothschildi* | Bali Myna |
| *Eos semilarvata* | Blue-eared Lory |
| *Pauxi unicornis* | Horned Curassow |
| *Ptilopachus nahani* | Nahan’s Partridge |
| *Leptotila battyi* | Brown-backed Dove |
| *Ptilinopus arcanus* | Negros Fruit-dove |
| *Amazona ventralis* | Hispaniolan Amazon |
| *Oriolus cruentus* | Javan Oriole |
| *Treron griveaudi* | Comoro Green-pigeon |
| *Bostrychia bocagei* | Dwarf Ibis |
| *Amazona brasiliensis* | Red-tailed Amazon |
| *Pauxi koepckeae* | Sira Curassow |
| *Columba junoniae* | White-tailed Laurel-pigeon |
| *Tinamus osgoodi* | Black Tinamou |
| *Scleroptila elgonensis* | Elgon Francolin |
| *Amazona rhodocorytha* | Red-browed Amazon |
| *Thaumatibis gigantea* | Giant Ibis |
| *Rheinardia ocellata* | Annamite Crested Argus |
| *Amazona guatemalae* | Northern Mealy Amazon |
| *Crypturellus boucardi* | Slaty-breasted Tinamou |
| *Eos histrio* | Red-and-blue Lory |
| *Amazona versicolor* | Saint Lucia Amazon |
| *Pipile pipile* | Trinidad Piping-guan |
| *Columba thomensis* | Sao Tome Olive-pigeon |
| *Geobiastes squamiger* | Scaly Ground-roller |
| *Treron sanctithomae* | Sao Tome Green-pigeon |
| *Trichoglossus rosenbergii* | Biak Lorikeet |
| *Oreophasis derbianus* | Horned Guan |
| *Brachypteracias leptosomus* | Short-legged Ground-roller |
| *Threskiornis bernieri* | Malagasy Sacred Ibis |
| *Atelornis crossleyi* | Rufous-headed Ground-roller |
| *Pampusana sanctaecrucis* | Santa Cruz Ground Dove |
| *Tanygnathus gramineus* | Black-lored Parrot |
| *Ducula mindorensis* | Mindoro Imperial-pigeon |
| *Ducula oceanica* | Micronesian Imperial-pigeon |
| *Gymnocrex talaudensis* | Talaud Rail |
| *Columba argentina* | Silvery Pigeon |
| *Geokichla leucolaema* | Enggano Thrush |
| *Ducula oenothorax* | Enggano Imperial-pigeon |
| *Hydrornis gurneyi* | Gurney's Pitta |
| *Accipiter butleri* | Nicobar Sparrowhawk |
| *Anthracoceros montani* | Sulu Hornbill |
| *Gallicolumba menagei* | Sulu Bleeding-heart |
| *Prioniturus verticalis* | Sulu Racquet-tail |
| *Phapitreron cinereiceps* | Tawitawi Brown-dove |
